# Leveraging Hybrid RF-VLP for High-Accuracy Indoor Localization with Sparse Anchors

**DOI:** 10.3390/s25103074

**Published:** 2025-05-13

**Authors:** Bangyan Lu, Yongyun Li, Yimao Sun, Yanbing Yang

**Affiliations:** 1 College of Computer Science, Sichuan University, Chengdu 610065, China; codeerror0x01@gmail.com (B.L.); lyyun@stu.scu.edu.cn (Y.L.); yimaosun@scu.edu.cn (Y.S.); 2 Institute for Industrial Internet Research, Sichuan University, Chengdu 610065, China

**Keywords:** visible light positioning (VLP), radio frequency positioning (RF-positioning), hybrid positioning, wireless sensor network (WSN)

## Abstract

Indoor low-power positioning systems have received much attention, and visible light positioning (VLP) shows great potential for its high accuracy and low power consumption. However, VLP also exhibits some limitations like small coverage area and the requirement of line of sight. Moreover, most VLP applications require the receiver to be within the coverage of at least three LEDs simultaneously, which seriously confines the availability of VLP when LEDs are sparsely deployed. Conversely, radio frequency (RF)-based positioning systems provide large coverage area, but suffer from low positioning accuracy due to multipath interference. In this work, we harnessed the complementary strengths of multiple technologies to develop a hybrid RF-VLP indoor positioning system for improving localization accuracy under sparse anchors. The RF-network-assisted visible light positioning enables each receiver to determine its position autonomously, using signals from a single LED anchor and neighboring receivers, and without needing RF anchors. To validate the effectiveness of the proposed method, we utilize commercial off-the-shelf LED and ESP32 to build up a prototype system. Comprehensive experiments are performed to evaluate the performance of the positioning system, and the results show that the proposed system achieves an overall root mean square error (RMSE) of 26.1 cm, representing a 28.5% improvement in positioning accuracy compared to traditional RF-based positioning methods, which makes it highly feasible for deployment.

## 1. Introduction

With the rapid growth of Internet of Things (IoT) technology, indoor positioning has shown extensive application prospects in smart homes, manufacturing, logistics, industrial automation and beyond [1]. Although global navigation satellite system (GNSS)-based outdoor positioning technology has become relatively mature, it is not well suited for indoor environments due to the signal attenuation caused by obstructions such as ceilings, walls, and other barriers [2]. Attaining precise positioning within indoor environments has emerged as a central focus in current research.

Indoor positioning can be categorized based on the different positioning media used, which mainly includes radio-frequency (RF)-based positioning [3] and visible light positioning (VLP) [4,5,6,7,8]. RF-based indoor positioning usually utilizes existing indoor Wi-Fi or Bluetooth signals as positioning media. They offer advantages like large coverage range, scalability, and ease of integration in indoor environments, making them popular in sectors such as retail and logistics. However, it also has limitations, including multipath interference and reduced accuracy in dynamic environments [9]. High infrastructure requirements and periodic maintenance for battery-powered beacons add to operational costs, while security vulnerabilities can impact data integrity. VLP on the other hand, offers high precision and immunity to radio-frequency interference, making it particularly effective in indoor environments where other signals may falter [10]. Since it uses existing LED lighting, VLP can easily integrate with the infrastructure, often reducing the installation costs and energy consumption, making it a promising option for indoor positioning applications [11,12,13,14,15,16]. However, VLP has limitations, including dependence on line-of-sight, which can disrupt signals in crowded spaces or if objects block the light source [17]. Furthermore, most VLP systems require the receiver to simultaneously acquire modulated optical signals from at least three LED anchors to achieve successful positioning [18,19,20], which seriously confines the availability of VLP when LEDs are sparsely deployed. Although some articles propose a single-LED anchor-based positioning system, a complex receiver design is required to achieve positioning [21,22], which increases the cost and complexity of the system. Focusing on applications such as factory automation, conveyors and robots usually need to perform localization under dynamic conditions. This process demands high levels of continuity, real-time responsiveness, and extensive coverage for target localization, rendering single localization methods insufficient to meet these complex requirements.

Since the RF and VLP systems are somewhat complementary to each other, the concept of a hybrid RF-VLP has become a new research trend. Albraheem et al. [23] proposed a BLE-VLC hybrid indoor positioning network, where BLE trilateration is fused with VLP distance information to obtain better positioning results. In [24], Singh et al. used the intensities of different wavelengths of light from various LED beacons and the signal strengths from different Bluetooth beacons to train a deep neural network (DNN). The trained model is then used to locate the target. Li et al. [25] used Wi-Fi and visible light RSS as fingerprints to achieve positioning, which was then applied to a simultaneous localization and mapping (SLAM) system. However, those existing positioning methods still require the positioning target within multiple anchors’ coverage area, which not only limits the size of the localization area but also necessitates that the receiver must have a wider field of view (FOV) to ensure signal reception.

To this end, we propose an advanced hybrid RF-VLP indoor positioning system that synergistically integrates the strengths of radio frequency and visible light technologies to overcome the inherent limitations of conventional methods. The proposed approach enables precise localization by utilizing a single photodetector (e.g., a photodiode or solar cell) to acquire signals from a single LED anchor, eliminating the need for multiple LEDs or fixed RF anchors. To enhance system efficiency and scalability, we incorporate a BLE mesh network to facilitate inter-receiver communication and provide auxiliary spatial information for positioning. This system combines VLP with BLE-based RF localization to address challenges faced by traditional positioning systems, such as low accuracy in RF localization and the dependence on multiple anchors in VLP systems. By reducing infrastructure requirements and enhancing system adaptability, this approach is well suited for deployment in complex indoor environments. Experiments show that this system achieves an overall root mean square error (RMSE) of 26.1 cm within an area of 1.93 m × 1.93 m, corresponding to an accuracy enhancement of approximately 28.5% over the traditional BLE-only localization approach. Furthermore, when compared to single LED-only positioning, an improvement of 74% is achieved due to the single LED anchor yielding localization error over 1 m.

This paper is organized as follows: Section 2 presents the system overview along with the proposed localization algorithm. Section 3 details the prototype design and experimental setup, while Section 4 offers an in-depth analysis of the experimental results. Finally, Section 5 concludes the paper and discusses potential future research directions.

## 2. Hybrid RF-VLP-Enabled Positioning System

### 2.1. System Overview

**Figure 1:**
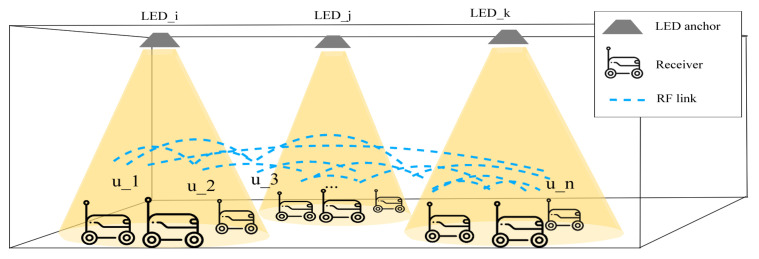
Components of the proposed positioning system.

As illustrated in Figure 1, the proposed system is a hybrid RF-VLP system, combining the benefits of RF and optical signals for robust indoor positioning. The system consists of LED anchors deployed on the ceiling, with non-overlapping coverage areas, and each LED broadcasts a unique ID. The receivers, placed on robots or automated guided vehicles, are equipped with RF transceivers, enabling communication with each other via a mesh network. The RF component of our system is based on BLE, but the system is designed for flexibility, enabling the use of other wireless technologies such as Wi-Fi or Zigbee depending on the specific requirements of the application, such as range, power efficiency, and network topology. Each receiver typically receives a signal from one LED at a time. The hybrid transceiver measures the received signal strength (RSS) of the optical signal to estimate the distance to the LED. Additionally, inter-receiver RSS measurements are used to estimate the relative distances between receivers. By combining these measurements, an integrated positioning algorithm computes the precise locations of all receivers. A potential application of this system could be in a smart building where accurate indoor navigation is essential. For example, in a large warehouse, this hybrid RF-VLP system could provide the real-time positioning of inventory and workers, improving operational efficiency and safety. Section 2.2 will describe the proposed localization algorithm in detail.

### 2.2. Integrated Localization Algorithm

**Figure 2:**
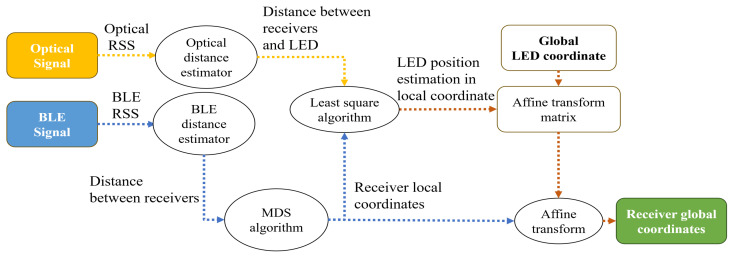
Overview of the proposed localization algorithm.

The overview of the localization algorithm is shown in Figure 2. The algorithm takes the RSS of both the optical and Bluetooth signals as input. After a series of computations, it outputs the global coordinates of the receiver. The optical signals are sourced from the LED anchors deployed indoors. We assume that there are M LED anchors installed on the ceiling. Each LED anchor is denoted as ${\mathrm{LED}}_{m},\left(m=1,\ldots ,M\right)$. Meanwhile, N (N≥3) receivers are below each of the LED anchors, sample the optical signal from the LED, and each of the receivers is denoted as ${\mathrm{Rx}}_{i},\left(i=1,\ldots ,N*M\right)$. Each pair of receivers is connected via a BLE link, enabling the estimation of distance between any two receivers through Bluetooth RSS. According to the Friis Equation [26], the received BLE power $P_{br}$ is equal to

(1) $$P_{br}=\frac{P_{bt}G_{t}G_{r}\lambda^{2}}{{\left(4\pi d_{b}\right)}^{2}},$$

where $P_{bt}$ is the BLE transmission power, $G_{t}$ and $G_{r}$ are the transmitter and receiver antenna gain, and λ is the signal wavelength in meter. By expressing power in decibels and performing transformations, $P_{br}$ can be represented as a logarithmic distributed random variable with a distance-dependent mean value, that is

(2) $$P_{br}=P_{br0}-10n_{p}\log_{10}\left(\frac{d_{b}}{d_{b0}}\right)+X_{\sigma },$$

where $P_{br0}$ is a reference power at a known distance $d_{b0}$ from the transmitter, $n_{p}$ is the propagation loss factor, and $X_{\sigma }$ is a zero mean Gaussian distributed random variable with standard deviation σ. Therefore, by collecting Bluetooth signal strength data at various distances and substituting it into Equation (2), the value of $n_{p}$ can be estimated and used for distance mapping in the localization process. The distance can be expressed as

(3) $$d_{b}=10^{\frac{P_{br0}-P_{br}}{10n_{p}}}d_{b0}.$$

Similarly, the distance between the LED anchor and the receiver can be estimated using the optical signal’s RSS. According to the Lambert emission law [27], the received optical signal power can be expressed as

(4) $$P_{or}=\frac{(s+1)A}{2\pi d_{o}^{2}}\cos^{s}\left(\theta \right)\cos\left(\phi \right)P_{ot},$$

where *A* represents the optical sensor surface area, θ and ϕ are the irradiance and incidence angle of LED and optical sensor, respectively. *s* is the Lambertian order of the LED, $P_{ot}$ is the LED’s transmission power. We assume that the light sensor of the receiver is oriented vertically upward, so θ is equal to ϕ. Then, Equation (4) can be simplified as

(5) $$P_{or}=\frac{\left(s+1\right)AP_{ot}}{2\pi d_{o}^{s+3}}h^{s+1},$$

where *h* represents the height of the LED anchor. Take a reference power $P_{or0}$ at a known distance $d_{o0}$, then according to Equation (5),

(6) $$\frac{P_{or}}{P_{or0}}={\left(\frac{d_{o0}}{d_{o}}\right)}^{s+3},$$

then *m* can be estimated and calibrated with the measured optical RSS value at different distance. After measuring the value of *m*, the distance between the LED and receiver can be expressed as

(7) do=do0PorPor0s+3.

Once the distance between each pair of receivers and the distance between each receiver and the LED are estimated, the coordinates of the receivers can be calculated using the following algorithm. Let $d_{bij}$ be the Euclidean distance between ${\mathrm{Rx}}_{i}$ and ${\mathrm{Rx}}_{j}$, then we can define a distance matrix as $\Delta ={\left[d_{bij}\right]}_{I\times I}$, where *I* represents the total number of receivers, $d_{bii}=0$, and $d_{bij}=d_{bji}$. Next, a local coordinate map containing all receivers can be established via a classical multi-dimensional scaling (MDS) algorithm [28]

(8) $$U_{\mathrm{local}}=\mathrm{MDS}\left(\Delta \right).$$

Note that $U_{\mathrm{local}}$ is a 2×N matrix that contains all receiver coordinates in the local 2D coordinate system. To determine the global coordinates of each receiver, we need to map this local coordinate to the absolute coordinate. To implement this remapping, we first estimate the LED coordinate in this local coordinate system. Assuming that ${\mathrm{Rx}}_{i}$ to ${\mathrm{Rx}}_{j}$ is within the coverage range of ${\mathrm{LED}}_{m}$, then the distance between ${\mathrm{LED}}_{m}$ and each receiver can be estimated using the optical RSS. We denote the distance between ${\mathrm{LED}}_{m}$ and ${\mathrm{Rx}}_{i}$ as $d_{oim}$. Suppose that the local coordinate of ${\mathrm{Rx}}_{i}$ is $U_{\mathrm{local}}\left(:,i\right)={\left(x_{i},y_{i}\right)}^{T}$, then the following equation can be constructed as

(9) $${\left(x_{m}-x_{i}\right)}^{2}+{\left(y_{m}-y_{i}\right)}^{2}+h^{2}=d_{oim}^{2},$$

where $l_{m}={\left(x_{m},y_{m}\right)}^{T}$ is the coordinate of ${\mathrm{LED}}_{m}$ that needs to be estimated in the local coordinate system. Then, this coordinate can be estimated via least square method

(10) $$l_{m}={\left(X^{T}X\right)}^{-1}X^{T}y,$$

where $X=\left[\begin{array}{cc} 2(x_{i}-x_{i+1}) & 2(y_{i}-y_{i+1})\\ 2(x_{i}-x_{i+2}) & 2(y_{i}-y_{i+2})\\ \vdots & \vdots \\ 2(x_{i}-x_{j}) & 2(y_{i}-y_{j}) \end{array}\right]$, $y=\left[\begin{array}{c} d_{o(i+1)m}^{2}-d_{oim}^{2}+\left(x_{i}^{2}+y_{i}^{2}\right)-\left(x_{i+1}^{2}-y_{i+1}^{2}\right)\\ d_{o(i+2)m}^{2}-d_{oim}^{2}+\left(x_{i}^{2}+y_{i}^{2}\right)-\left(x_{i+2}^{2}-y_{i+2}^{2}\right)\\ \vdots \\ d_{ojm}^{2}-d_{oim}^{2}+\left(x_{i}^{2}+y_{i}^{2}\right)-\left(x_{j}^{2}-y_{j}^{2}\right) \end{array}\right]$.

Apply Equation (10) to each LED, so we can establish a matrix of LED local coordinate $L_{local}=\left[l_{1},l_{2},\ldots ,l_{M}\right]$.

Upon constructing the matrix $L_{\mathrm{local}}$, the global coordinates of each receiver can be obtained by transforming the local coordinates into the absolute coordinate system. Assume that the absolute coordinates of each LED anchor is $L_{\mathrm{abs}}=\left[l_{1\_\mathrm{abs}},l_{2\_\mathrm{abs}},\ldots ,l_{M\_\mathrm{abs}}\right]$, then the local coordinate can be converted into the absolute coordinate via affine transformation. Let $L_{\mathrm{local}}^{\prime }=\left[\begin{array}{c} L_{\mathrm{local}}\\ 1_{1\times M} \end{array}\right]$, and $L_{\mathrm{abs}}^{\prime }=\left[\begin{array}{c} L_{\mathrm{abs}}\\ 1_{1\times M} \end{array}\right]$, then the absolute coordinates can be expressed as

(11) $$L_{\mathrm{abs}}^{\prime }=A*L_{\mathrm{local}}^{\prime },$$

where A represents the transform matrix. And we can then convert the local receiver coordinates to global coordinates

(12) $$U_{\mathrm{abs}}^{\prime }=A*U_{\mathrm{local}}^{\prime }$$

where $U_{\mathrm{local}}^{\prime }=\left[\begin{array}{c} U_{\mathrm{local}}\\ 1_{1\times M} \end{array}\right]$, and $U_{\mathrm{abs}}=U_{\mathrm{abs}}^{\prime }\left(1:2,:\right)$.

## 3. Prototype and Experimental Setup

**Figure 3:**
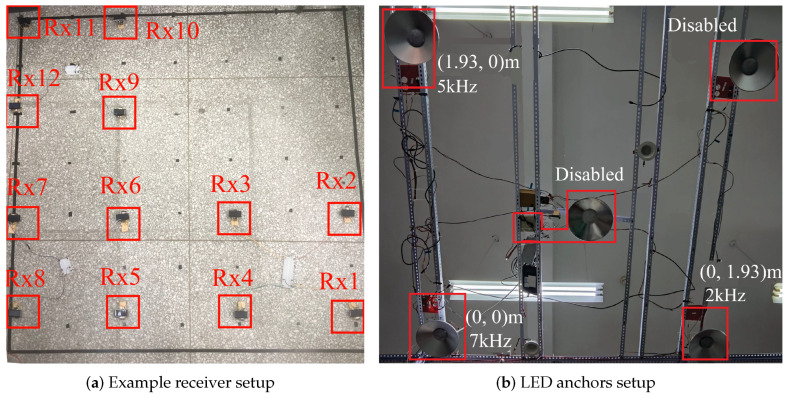
LED anchor and example receiver setup.

In order to evaluate the performance of the proposed localization system, we build a prototype system within a controlled indoor environment measuring 1.93 m × 1.93 m × 2.57 m. The system includes three commercial off-the-shelf (COTS) white LEDs acting as positioning anchors, mounted on the ceiling at coordinates $l_{1}=\left(0,0\right)$ m, $l_{2}=\left(0,1.93\right)$ m and $l_{3}=\left(1.93,0\right)$ m, all at a height of h=2.57 m (see Figure 3b). Each LED transmits a modulated optical signal at a different frequency, where $f_{1}=7\;\mathrm{kHz}$, $f_{2}=2\;\mathrm{kHz}$, $f_{3}=5\;\mathrm{kHz}$. Note that despite the fact that there is an existing overlap of LED coverage due to the spatial constraints of our laboratory, each receiver uses frequency division to utilize signals from a single LED.

We deploy 12 receivers on the ground, organized into three groups corresponding to the coverage areas of the three LEDs, with each group containing four receivers (Figure 3a). The receiver hardware is constructed using commercial ESP32-S3 microcontrollers (MCUs) producted by Espressif Systems, Shanghai, China. A solar panel measuring 37 mm × 68 mm serves as the light sensor for each receiver. The receiver circuit converts the optical current into voltage signal and feeds it to the ESP32-S3 MCU. For signal processing, the analog-to-digital converter (ADC) of the ESP32-S3 samples the optical signal at a frequency of fs=20Ksps. We apply a fast Fourier transform (FFT) to the sampled data to extract the amplitude of each LED’s signal, which is then used to estimate the distance between the receiver and the corresponding LED anchor.

**Figure 4:**
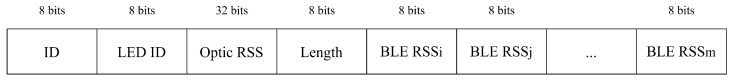
BLE mesh vendor message structure.

**Figure 5:**
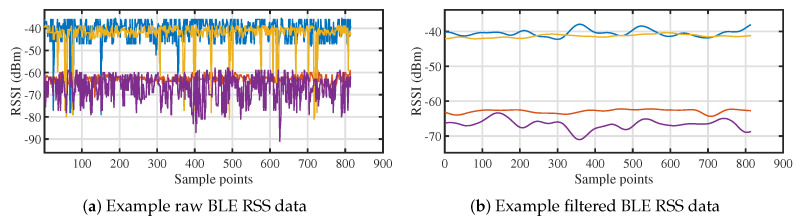
Comparison of BLE RSS metrics pre- and post-filtering.

Regarding the Bluetooth functionality, the ESP32-S3’s 2.4 GHz wireless interface is employed for BLE communication and ranging. Each receiver operates as a BLE mesh node, sharing a common network key to facilitate secure communication. We implement custom BLE vendor server and client models on each receiver. The vendor client periodically broadcasts messages containing the receiver’s ID, associated LED anchor ID, optical RSS, and a list of BLE RSS measurements between itself and other receivers ($\mathrm{BLE}\;{\mathrm{RSS}}_{m}$ for receiver ${\mathrm{Rx}}_{m}$), as illustrated in Figure 4. The message’s time-to-live (TTL) is set to zero to prevent forwarding. The vendor server on each receiver collects incoming messages and stores the pertinent information for localization processing. The raw BLE RSS measurements collected between receivers are shown in Figure 5a. It is evident from the data that the raw signal contains noticeable outliers and Gaussian noise, which can be attributed to interference from the hardware components as well as noise from the 2.4 GHz signal band. To mitigate the impact of this noise, we apply both a Hample filter and a Gaussian filter to the raw signal. The filtered RSS data, shown in Figure 5b, demonstrate a significant reduction in noise, resulting in a cleaner and more stable signal. This preprocessing step is essential for improving the accuracy of subsequent positioning calculations by reducing the influence of undesirable signal fluctuations and external interferences.

## 4. Results and Discussions

### 4.1. Comparison Among Different Positioning Systems

**Figure 6:**
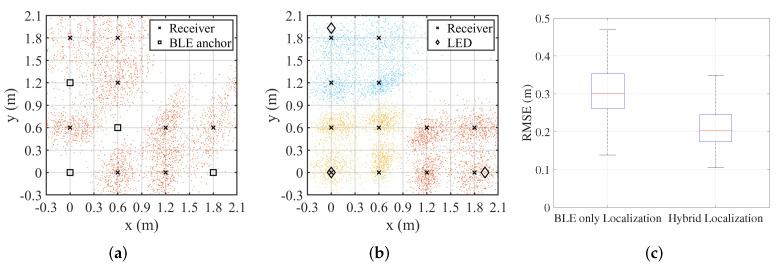
Comparison with BLE-only positioning systems. (**a**) BLE only localization. (**b**) Hybrid localization. (**c**) RMSE of positioning for all receivers.

To highlight the advantages of the hybrid system, comparisons are made with the widely used BLE-only positioning systems. In this configuration, all LED anchors are disabled, and four receivers are randomly selected as anchors. The remaining receivers estimate their distances to the anchors using Equation (3) and compute their coordinates with the least squares method. The receiver setup and the positioning result are shown in Figure 6a. And, as shown in Figure 6c, the traditional BLE-only system achieves an overall root mean square error (RMSE) of 36.5 cm. In contrast, the proposed hybrid system demonstrates a notable improvement. The receiver setup and positioning result are shown in Figure 6b, and dots of different colors represent the positioning results of receivers within the coverage areas of different LEDs. The positioning result achieves an overall RMSE of 26.1 cm, corresponding to an accuracy enhancement of approximately 28.5% over the BLE-only localization approach.

Another widely used localization method is LED-only positioning. However, due to the non-overlapping coverage areas of LED anchors, location estimation is limited to proximity-based techniques. Consequently, the localization error is approximately equal to the LED coverage radius, around 1 m, which is significantly worse than the proposed method.

### 4.2. The Effect of BLE Transmission Power on Hybrid System Localization Accuracy

**Figure 7:**
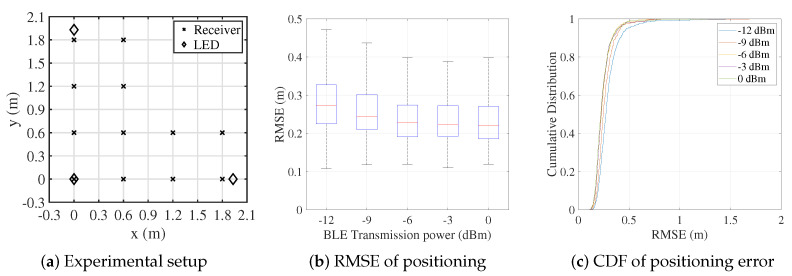
Localization error of the hybrid system under different BLE transmission power levels.

The proposed system utilizes BLE signals for inter-receiver distance estimation and local coordinate construction. To assess the impact of BLE transmission power on ranging and positioning performance, we systematically test the system under varying power levels. Changes in transmission power directly influence the signal strength and communication range between receivers, enabling a controlled evaluation of the trade-offs between power consumption, communication range, and positioning accuracy. For the experiments, 12 receivers are deployed at fixed positions within the test area, as illustrated in Figure 7a, alongside the LED anchors. The BLE transmission power is adjusted across all receivers, and the resulting localization errors are analyzed. The proposed hybrid positioning method is applied, with the experimental results presented in Figure 7. It can be observed that the positioning error decreases as the BLE transmission power increases. However, once the transmission power exceeds −3 dBm, the improvement in positioning accuracy becomes marginal, indicating diminishing returns beyond this point.

Higher transmission power enhances the signal coverage and signal-to-noise ratio, reducing the communication bit error rate and further improving the positioning accuracy. Considering the trade-off between localization error and system power consumption, −3 dBm is selected as the optimal BLE transmission power for subsequent experiments.

### 4.3. Impact of Receiver Arrangement on Localization Accuracy

**Figure 8:**
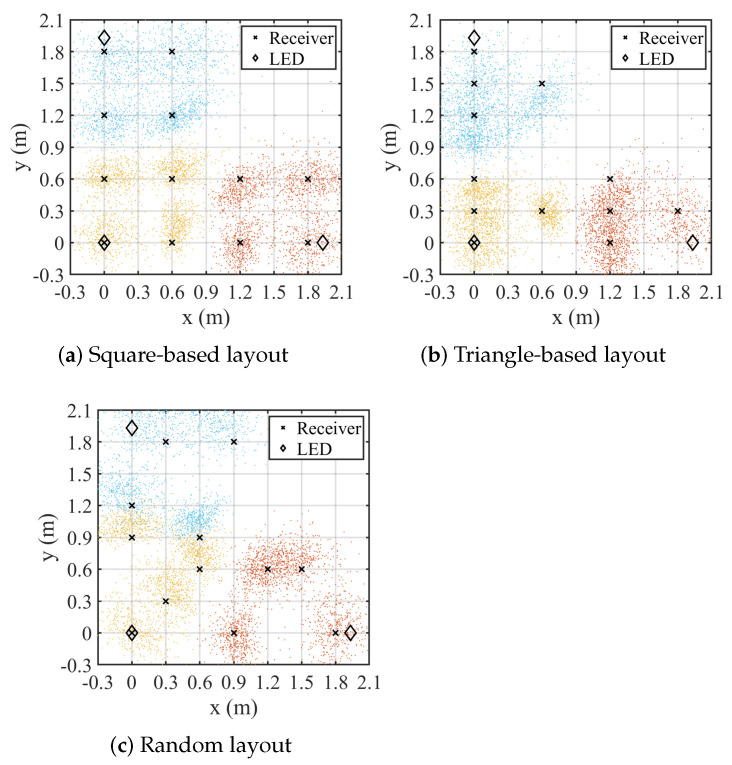
Localization result under different receiver arrangement.

**Figure 9:**
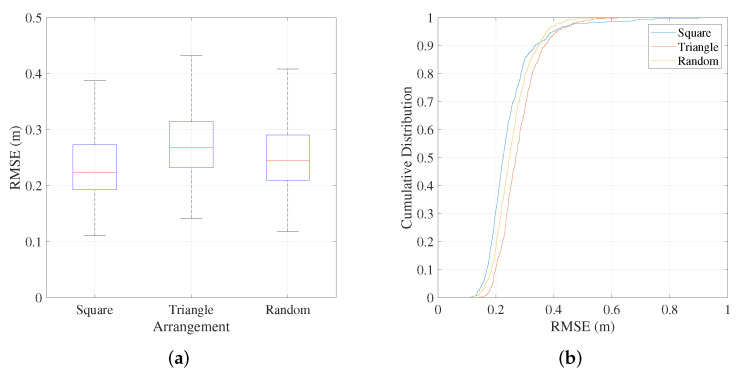
Localization error under different receiver arrangement. (**a**) RMSE under different receiver arrangement. (**b**) CDF under different receiver arrangement.

To verify the versatility of the positioning system in real-world environments, we analyze its positioning performance under different receiver arrangements. The detailed arrangements are shown in Figure 8, square and triangle receiver arrangements are tested as two typical scenarios. And, to better demonstrate localization accuracy in more general scenarios, we also include a random receiver arrangements and evaluate their localization accuracy. In each experiment, 12 receivers are positioned at a fixed coordinate, and the BLE transmitting power is set to −3 dBm. A total of 500 experiments are conducted for each arrangement. The positioning results of each experiment are shown in Figure 8, with different colored dots representing the positioning results of receivers within the coverage areas of different LEDs, and the positioning error is shown in Figure 9. Overall, the RMSE of the positioning accuracy for all nodes under the square arrangement is 26.1 cm, with an average error of 21.7 cm. Additionally, it is observed that the positioning results for receivers closer to the center of the positioning area are more accurate, while those near the edges are more dispersed. This is due to the presence of walls and other obstructions at the edges, which interfere with Bluetooth and optical signals, leading to a lower signal-to-noise ratio and a slight increase in positioning error in those areas. Under different receiver arrangements, the square array exhibits the best localization performance. Although the localization accuracy under random arrangement is slightly lower than that of the square array, it still achieves an average localization error of approximately 23 cm. In the triangular arrangement, the proximity of receivers to each other lead to interference from Bluetooth signals, resulting in relatively lower localization accuracy. Nonetheless, in typical application scenarios, the receivers are often randomly distributed, yet reliable localization accuracy remains achievable.

### 4.4. Impact of Receiver Spacing on Localization Accuracy

**Figure 10:**
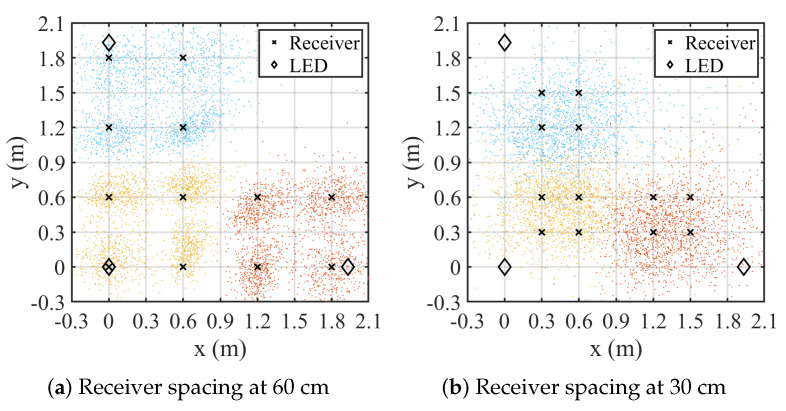
Localization result under different receiver spacing.

The impact of receiver spacing on localization performance is further analyzed. The experimental setup is depicted in Figure 10. The square receiver arrangement, which achieves the highest accuracy in previous experiments, is employed for this analysis. Reducing the square side length from 60 cm to 30 cm significantly increases the mean positioning error to 64 cm, highlighting a drop in localization accuracy. The closer receiver spacing results in a higher geometric dilution of precision (GDOP) [29] when applying Equation (10) for positioning, leading to greater errors in LED coordinate estimation. Additionally, excessively close receiver spacing causes BLE ranging errors to contribute disproportionately to the overall ranging results, further degrading the computational accuracy of the MDS algorithm. When BLE ranging is not significantly affected by distance-related interference, larger receiver spacing improves the localization accuracy. In practical localization scenarios, where receivers are typically randomly distributed, this condition is often naturally satisfied.

### 4.5. Summary of Experimental Results

The proposed hybrid RF-VLP positioning system outperforms the traditional BLE-only system, achieving a 28.5% improvement in localization accuracy with an RMSE reduction from 36.5 cm to 26.1 cm. Since the receiver layout in this positioning system is random, we verify the system’s positioning performance under different receiver arrangements. Experiments show that the square arrangement yields the best results (RMSE of 26.1 cm), while random and triangular configurations still maintain reliable accuracy, averaging around 23 cm. Although smaller receiver spacing can lead to poorer positioning accuracy due to increased GDOP, the proposed system remains highly effective in large-scale positioning scenarios, where receiver spacing is typically larger. In these scenarios, the system continues to deliver accurate results, making it suitable for practical, real-world applications.

## 5. Conclusions

The paper presents a hybrid RF-VLP positioning system that integrates an RF-assisted VLP algorithm, enabling each receiver to accurately determine its location using the signals from a single LED anchor without relying on additional fixed RF anchors. A prototype system is developed and its positioning performance is analyzed across various environments. The experimental results reveal that the system achieves a positioning RMSE of approximately 26.1 cm, outperforming traditional Bluetooth-based positioning solutions under comparable conditions. Furthermore, we analyze the positioning performance under different receiver configurations, and the results indicate that the system consistently achieves decimeter-level accuracy across various configurations, demonstrating its versatility. The proposed RF-VLP hybrid positioning approach effectively addresses the limitations of conventional methods by enhancing the low accuracy of RF-based systems and extending the limited range of traditional VLP solutions. In summary, the system proposed in this paper offers a scalable and promising solution for future indoor positioning applications, such as intelligent logistics. In the future, we will also discuss and conduct further research on more complex positioning scenarios.

## Data Availability

The original contributions presented in this study are included in the article. Further inquiries can be directed to the corresponding author.
